# Puf6 and Loc1 Are the Dedicated Chaperones of Ribosomal Protein Rpl43 in *Saccharomyces cerevisiae*

**DOI:** 10.3390/ijms20235941

**Published:** 2019-11-26

**Authors:** Kai-Jen Liang, Le-Yun Yueh, Ning-Hsiang Hsu, Jui-Sheng Lai, Kai-Yin Lo

**Affiliations:** Department of Agricultural Chemistry, National Taiwan University, Taipei 10617, Taiwan

**Keywords:** ribosome biogenesis, ribosomal proteins, transacting factors, chaperone

## Abstract

Ribosomal proteins are highly expressed, and the quality of ribosomal proteins must be rigorously controlled to build up a functional ribosome. Rpl43, ribosomal protein large subunit 43, is located nearby the E-site of ribosomes. In our previous study, we found that Puf6, Loc1, and Rpl43 form a trimeric complex in *Saccharomyces cerevisiae*. Rpl43 protein levels are under-accumulated in the absence of *PUF6* or *LOC1*. However, why the loss of Puf6 or Loc1 decreased the protein levels of Rpl43 remained unclear. In the present study, we further dissected the connections among these three proteins and found that the processing defects of pre-ribosomal RNA in *puf6*Δ and *loc1*Δ are similar to those of the mutant with depletion of Rpl43. The stability of newly synthesized Rpl43 protein decreased slightly in *puf6*Δ and significantly in *loc1*Δ. We also found that Puf6 and Loc1 could interact with nascent Rpl43 co-translationally via the N-terminus of Rpl43. While the association and dissociation of Rpl43 with karyopherins did not depend on Puf6 and Loc1, Puf6 and Loc1 interacted with nascent Rpl43 in collaboration. While the N-terminus of Puf6 contained nuclear localization signals for transport, the PUF (Pumilio) domain was essential to interaction with Loc1, Rpl43, and 60S subunits. The C-terminus of Loc1 is more important for interaction with Puf6 and Rpl43. In this study, we found that Puf6 and Loc1 are the dedicated chaperones of ribosomal protein Rpl43 and also analyzed the potential interaction domains among the three proteins. Correct formation of the Puf6, Loc1, and Rpl43 ternary complex is required to properly proceed to the next step in 60S biogenesis.

## 1. Introduction

Ribosomes are massive complexes composed of ribosomal proteins and RNAs. Ribosome biogenesis is necessary for cell growth and proliferation and represents one of the major energy cost pathways of cells. Rapidly growing yeast cells generate about 2000 ribosomal subunits per minute [1]. Indeed, about 60% of the total transcription in a cell is devoted to ribosomal RNA synthesis to support such large-scale synthesis. The multiple steps of ribosome biogenesis allow cells to precisely regulate the balance between supply and demand. Cells can regulate at the RNA transcriptional and ribosomal protein synthesis levels [1,2,3,4].

Over 200 transacting (non-ribosomal) factors have been identified in the ribosome assembly process; these factors present various and important functions, such as maintenance of the stability of nascent ribosomal proteins, functional proofreading of ribosomes, assurance of correct interaction between ribosomal proteins and rRNAs, and processing and folding of rRNAs. Transacting factors join in the ribosome synthesis process sequentially to produce ribosomes assembled through a hierarchical mechanism (see refs [5,6,7,8,9,10,11]). Ribosomes in yeast are composed of 79 ribosomal proteins encoded by 138 ribosomal protein genes. Many of the ribosomal proteins are encoded by two genes to generate isoforms with identical or highly similar amino acid sequences [12]. To generate this enormous amount of ribosomal proteins, cells in the logarithmic phase devote approximately 50% of their total transcription activities of the RNA polymerase II [13].

Whereas ribosomes have a very long half-life, ribosomal proteins are very unstable, likely because of their structure, which features positive charges and long unstructured extensions [14]. Therefore, unincorporated ribosomal proteins could be degraded rapidly [15]. Cells adapt common chaperones and importins to provide general protection to ribosomal proteins. Cells can even evolve a specialized chaperone system to interact with ribosomal proteins specifically. These dedicated chaperones could regulate the stability, transport, stoichiometry, and orientation of ribosomal proteins during the assembly process [14,16,17,18,19,20,21,22,23,24,25,26,27,28,29,30,31,32,33].

Ribosomal protein large subunit 43 (Rpl43) and its human homolog, L37A, have been renamed L43e in the new nomenclature system [34]. Rpl43 is essential and uniquely present in Archaea and eukaryotes but not in prokaryotes. Rpl43 is a small ribosomal protein in the large subunit with only 94 amino acids. It is located in the vicinity of the E-site of 60S subunits and in close contact with Rpl2 **[35]**. Rpl2 and Rpl43 are found close to the peptidyl transferase center and required for 7S rRNA production [36]. Two homologs of Rpl43 have been identified in yeast, namely, *RPL43A* and *RPL43B*, and these homologs share 90% identity in DNA sequence and 100% identity in amino acid sequence. Puf6 and Loc1 have previously been shown to recognize the 3′ untranslated region (UTR) and control the asymmetric localization of *ASH1* mRNA in yeast [37]. In our previous study, we found that Puf6 and Loc1 form a complex with Rpl43. In the absence of Puf6 or Loc1, the loading and protein levels of Rpl43 are decreased [38]. Puf6 and Loc1 are loaded onto 60S together with Rpl43. If Rpl43 is depleted, no effect on loading is observed, but the release of Puf6 and Loc1 is impaired. Thus, recruitment of Rpl43 is required for the release of Puf6 and Loc1 [38]. However, the detailed molecular mechanism behind these events remains unclear. In the present work, we found that Puf6 and Loc1 are critical to maintaining the stability of Rpl43. Mutation of any one protein results in blockages at similar steps during biogenesis. We further dissect how these three proteins interact with each other and conclude that Puf6 and Loc1 are the dedicated chaperones of Rpl43.

## 2. Materials and Methods

### 2.1. Strains, Plasmids, and Reagents

All *Saccharomyces cerevisiae* strains used in this study are listed in Table 1. Unless otherwise indicated, all strains were grown at 30 °C in rich medium (yeast extract peptone) or synthetic dropout medium containing 2% glucose (Glc). The plasmids used in this study are listed in Table 2.

Anti-Loc1, anti-Puf6, anti-Tif6, anti-Rpl23, anti-Rpl8 [38], and anti-Rpl43 antibodies were generated in the lab. Anti-myc antibody was obtained from MYC 1-9E10.2 [9E10] (ATCC^®^ CRL1729™). Anti-HA (hemagglutinin) (MMS-101P), anti-TAP (Tandem affinity purification) (CAB1001), and anti-GST (glutathione S-transferase) (GST001M) were purchased from Covance, Thermo, and Bioman, respectively. Proteins were resolved in SDS-PAGE and transferred to a membrane with Trans-Blot^®^ SD Semi-Dry (BioRad). Signals were detected using Clarity^TM^ Western ECL substrate (Biorad, Hercules, CA, USA) and scanned by MultiGel-21 (Top Bio, Taipei, Taiwan). The signal intensity was by measured by Image J for quantification.

### 2.2. The Analysis of Protein Stability and Western Blotting

Yeast cells were grown in liquid culture until OD_600_ ~ 0.5, cycloheximide (200 μg/mL) was added, and the cells were harvested after a 10 min interval. The cell lysates were normalized and the samples were centrifuged for 1 h at 4 °C and 80,000 rpm in an MLA130 rotor (Beckman, Indianapolis, IN, USA) to separate the ribosomes and nascent (unincorporated) ribosomal proteins to the pellets and supernatant, respectively. The pellets (ribosome fraction) were re-dissolved in 1× Laemmli buffer. The proteins in the supernatant (free form) were precipitated by TCA and re-dissolved in 1× Laemmli buffer. The proteins were detected and analyzed by Western blotting.

### 2.3. Immunoprecipitation

Cultures were grown to an OD_600_ of ~0.5 in selective medium. For the preparation of protein extracts, cells were resuspended in IP buffer (20 mM Tris pH 7.5, 50 mM NaCl, 6 mM MgCl_2_, 10% glycerol, 1 mM PMSF (phenylmethylsulfonyl fluoride), and 1 mM leupeptin), lysed by vortexing with glass beads, and clarified by centrifugation. α-c-myc antibody (9E10) and protein-A agarose beads (GE Healthcare, Chicago, IL, USA) were added to normalized samples and rocked at 4 °C. The beads were washed three times with IP buffer and the proteins were eluted in 1× Laemmli sample buffer. Subsequently, proteins were separated in SDS-PAGE and observed by Western blotting.

To identify whether Puf6 and Loc1 associate with nascent Rpl43 co-translationally, we followed the procedures described in the previous study [32]. Briefly, cells were treated with cycloheximide and lysed in IP buffer containing RNasin^®^ ribonuclease inhibitors (Promega, Madison, WI, USA). IgG (Immunoglobulin G) sepharose beads (GE Healthcare) were added to immunopurify TAP-Tag proteins. After washes, samples were eluted in elution buffer (100 mM Tris pH 8, 10 mM EDTA, 1% SDS) and RNA was extracted using TRI reagent (Sigma, St. Louis, MO, USA). cDNA was synthesized using Reverse Transcriptase (Ambion, city, Waltham, MA, USA). The levels of translating *RPL43* mRNA were determined by quantitative real-time PCR (Applied Biosystems, Waltham, MA, USA) with Power SYBR Green (Thermo, Waltham, MA, USA) detection. *RPL5* and *RPL10* mRNA were included as controls.

### 2.4. Polysome Profile Analysis

Yeast cells were grown to an OD_600_ of about 0.2–0.3. Cycloheximide was added to a final concentration of 50 μg/mL and incubated continuously for 10 min. Cells were lysed with polysome lysis buffer (20 mM Tris HCl pH7.5, 8 mM MgCl_2_, 12 mM mercaptoethanol, 100 mM KCl, 50 μg/mL cycloheximide, 1 mM PMSF, 1 mM leupeptin), and nine OD_260_ units of protein extracts were loaded onto linear 7–47% sucrose gradients. After 2.5 h of centrifugation at 4 °C and 40,000 rpm in an SW41 Ti rotor (Beckman, Indianapolis, IN, USA), gradient fractions were continuously measured for their absorbance at 254 nm and sucrose fractions were collected (BR-188, Brandel, Gaithersburg, MD, USA). For detection of the RNA distribution, fractions were precipitated with 500 μL Trizol and 200 μL chloroform to extract RNA, then isopropanol was added to precipitate. The levels of RNA were quantitated by qPCR.

### 2.5. Northern Blotting

Northern blotting was used to analyze the static state levels of pre-rRNAs. The total RNA prepared with TRI reagent (Sigma, St. Louis, MO, USA) was resolved on a formaldehyde agarose gel for large-molecular-weight rRNAs or urea polyacrylamide gel for small-molecular-weight rRNAs. The RNAs were transferred to a nitrocellulose membrane. The probes were labeled with a Biotin 3ʹend labeling kit (Thermo, Waltham, MA, USA) and continually hybridized and detected with North2South^®^ Chemiluminescent hybridization and detection kits (Thermo), respectively. Table 3 lists the probe sequences.

### 2.6. In Vitro Interaction Experiments

Various *PUF6*, *LOC1*, and *RPL43* mutants were cloned into the pET28a or pGEX-4T3 vectors and overexpressed in *Escherichia coli* BL21 (DE3) as recombinant proteins with His_6_ or GST tags. Cell extracts were prepared by sonication (Chrom Tech, Taipei, Taiwan) in TEN100 lysis buffer (20 mM Tris, pH 7.4, 0.1 mM EDTA, and 100 mM NaCl). Whole-cell extracts containing GST or GST-tagged proteins were first incubated with glutathione beads for 1 h at 4 °C and washed with chilled TEN100 buffer five times. Protein extracts containing prey protein were subsequently incubated with glutathione beads for 1 h at 4 °C. Beads were washed with buffer five times and eluted with 1× SDS sample buffer. Samples were separated in SDS-PAGE and stained with Coomassie blue. The interacting proteins were further confirmed with anti-Puf6, anti-Loc1, and anti-GST antibodies in Western blotting.

## 3. Results

### 3.1. Mutations of Puf6, Loc1, and Rpl43 Show Similar rRNA Processing Defects

**Figure 1:**
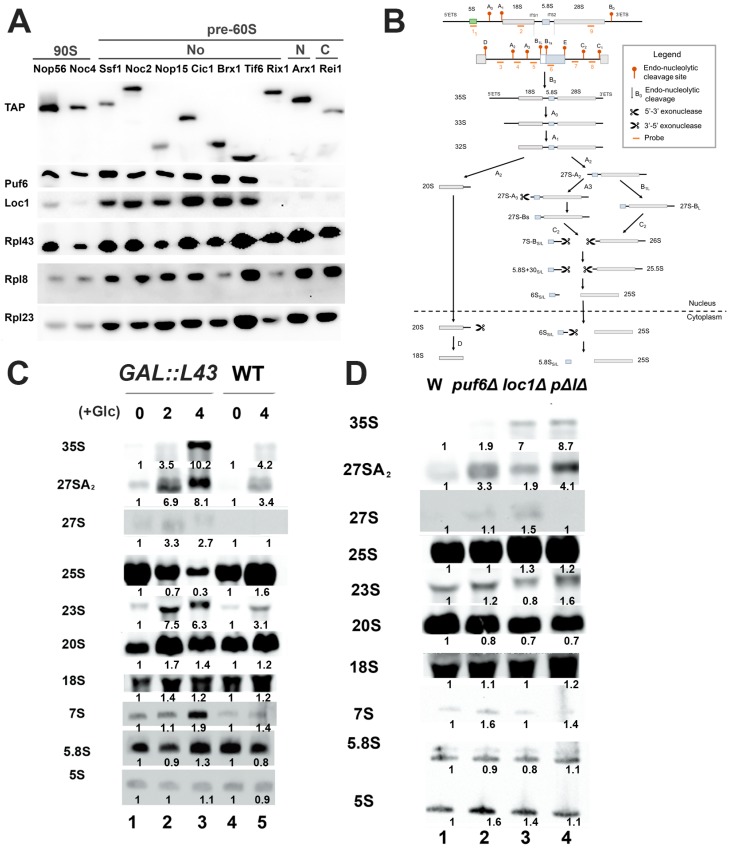
Mutation of either Puf6, Loc1, or Rpl43 showed similar rRNA processing defects. (**A**) Pre-90S and pre-60S ribosomal subunits at different stages were immunoprecipitated and detected by Western blotting. (**B**) The rRNA processing pathway and the probes used in the Northern blotting. (**C**,**D**) The rRNA processing was detected in the strains below with Northern blotting. (**C**) Wildtype (WT) and *GAL::RPL43* strains were cultured in the medium containing galactose. Glucose was added to stop the transcription of the *GAL*-driven promoter to shut down the expression of Rpl43. (**D**) WT, *puf6*Δ (KLY67), *loc1*Δ (KLY218), and *puf6*Δ*loc1*Δ (KLY312) were cultured at 30 °C until OD = 0.4~0.6. The cells were collected for RNA extraction. (**C**,**D**) The quantifications are shown below each sample. The relative intensities compared to initial state (**C**) and WT strain (**D**) were calculated.

In our previous study, Rpl43 formed a complex with Puf6 and Loc1 [38]. Moreover, deletion of Puf6 and Loc1 impacted the protein level and loading of Rpl43 onto 60S [38]. Pre-90S and pre-60S ribosomal subunits were immunoprecipitated by transacting factors at different stages to examine whether they are loaded onto the corresponding ribosomal subunits at similar stages (Figure 1A). Nop56 and Noc4 were used for purification of pre-90S; Ssf1, Noc2, Nop15, Cic1, Brx1, and Tif6 were used for purification of early pre-60S from the nucleolus to the nucleus; Rix1 and Arx1 were used for purification of late pre-60S in the nucleus; and Rei1 was used for purification of pre-60S in the cytoplasm [5,7,40,41,42]. Puf6, Loc1, and Rpl43 could be detected at the 90S stage. However, the major peak of these three proteins in pre-60S was observed at the nucleolar stage. Puf6 and Loc1 were not present at Rix1, Arx1, and Rei1. Although depletion of Rpl43 majorly influences the late stage of 7S rRNA processing [43], it has been shown to copurify with 35S, 32S, and 27S rRNAs [44]. This is consistent with the finding that Rpl43 is loaded onto the 90S pre-ribosome.

The status of rRNA processing in *rpl43* mutated, *puf6*Δ, and *loc1*Δ strains was evaluated. The probes used to detect the intermediates of rRNA processing are shown in Figure 1B. While deletion of *RPL43A* or *RPL43B* resulted in slight growth defects, deletion of both homologs caused lethality [45]. *RPL43* was constructed under a *GAL-*driven promoter and complemented in an *rpl43A*Δ*rpl43B*Δ strain. When glucose was added to this system to switch off the gene expression of *GAL::RPL43*, accumulation of 35S, 27S, 27SA2, and 23S was detected, which may be due to the blockage of C2 cleavage (Figure 1C). The mutants *puf6*Δ and *loc1*Δ also showed accumulation of 35S, 27S, and 27SA2 (Figure 1D). Therefore, Puf6, Loc1, and Rpl43 may participate at similar stages of ribosome biogenesis.

### 3.2. Puf6 and Loc1 Are Important for the Protein Stability of Rpl43

Ribosomal proteins are highly unstable because their structures feature highly positive charges and disordered extensions [14]. These extensions are inserted into ribosomes for contact with rRNAs and other ribosomal proteins to coordinate the conformational changes of the giant complex [14]. Several dedicated chaperones have recently been identified to maintain the protein stability of ribosomal proteins [14]. Since Puf6 and Loc1 could form a trimeric complex with Rpl43 (Figure 1 and [38]), we tested how Puf6 and Loc1 impacted the protein levels of Rpl43.

**Figure 2:**
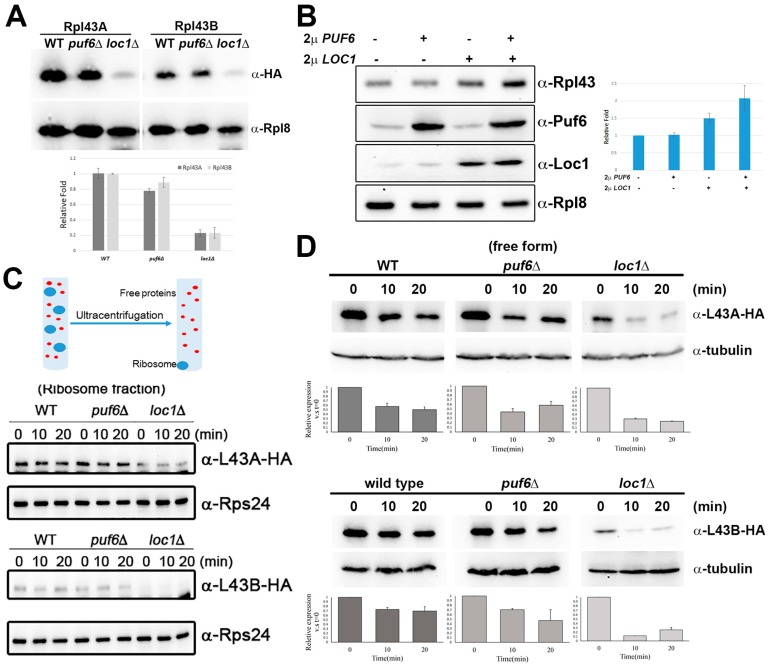
Puf6 and Loc1 are important for the protein stability of Rpl43. (**A**) The protein levels of Rpl43A-HA (PKL349) and Rpl43B-HA (PKL350) in wildtype (BY4741), *puf6*Δ (KLY67), and *loc1*Δ (KLY218) were detected by Western blotting. The signals were quantitated by Image J. HA signals were normalized with Rpl8, and the relative ratios to WT were calculated. (**B**) 2μ *PUF6* (PKL333) and *LOC1* (PKL746) were transformed to wild-type cells containing *RPL43B-HA* (PKL563). The protein levels were checked by Western blotting. The relative ratios of Rpl43 in *PUF6-* or *LOC1-* overexpression strains to the vector-only control were calculated. (**C**,**D**) Cycloheximide was added to the cells and incubated for different times. Cell lysates were spun at 80,000 rpm for 60 min at 4 °C. The pellets (ribosome fraction) were re-dissolved in 1× Laemmli buffer. The proteins in the supernatant (free form) were precipitated by TCA and re-dissolved in 1× Laemmli buffer. The proteins were detected by anti-tubulin and anti-HA antibodies. The signals were quantitated by Image J.

The levels of Rpl43A and Rpl43B were examined in *puf6*∆ and *loc1*∆ cells to analyze how Puf6 and Loc1 affect the protein level of Rpl43. While Rpl43A and Rpl43B decreased slightly in *puf6∆*, both decreased significantly in *loc1*∆ cells (Figure 2A). Overexpression of Puf6 or Loc1 alone did not or slightly increased the protein level of Rpl43, respectively. Overexpression of Puf6 and Loc1 together increased the protein levels of Rpl43 about 2.1-fold (Figure 2B).

The protein stability of nascent Rpl43 was further tested in *puf6*∆ and *loc1*∆. Cycloheximide was added to inhibit protein synthesis, and samples were collected at various time points. The majority of the ribosomal proteins are incorporated into mature ribosomal subunits and have long half-lives. Therefore, samples were spun on the ultracentrifuge to separate nascent ribosomal proteins into their free form and ribosomal subunits into pellets (Figure 2C, upper panel). Within the 20-minute treatment, the levels of Rpl43A and Rpl43B in the ribosome fractions remained constant (Figure 2C, lower panel). In contrast, the stability of nascent Rpl43 proteins was decreased significantly in *puf6∆* and *loc1*∆ (Figure 2D). The data above suggest that Puf6 and Loc1 are the chaperones of Rpl43, important for maintaining the protein stability of newly synthesized Rpl43.

### 3.3. Puf6 and Loc1 Can Associate with Nascent Rpl43 but Are Not Required for Kap Interaction or Release

**Figure 3:**
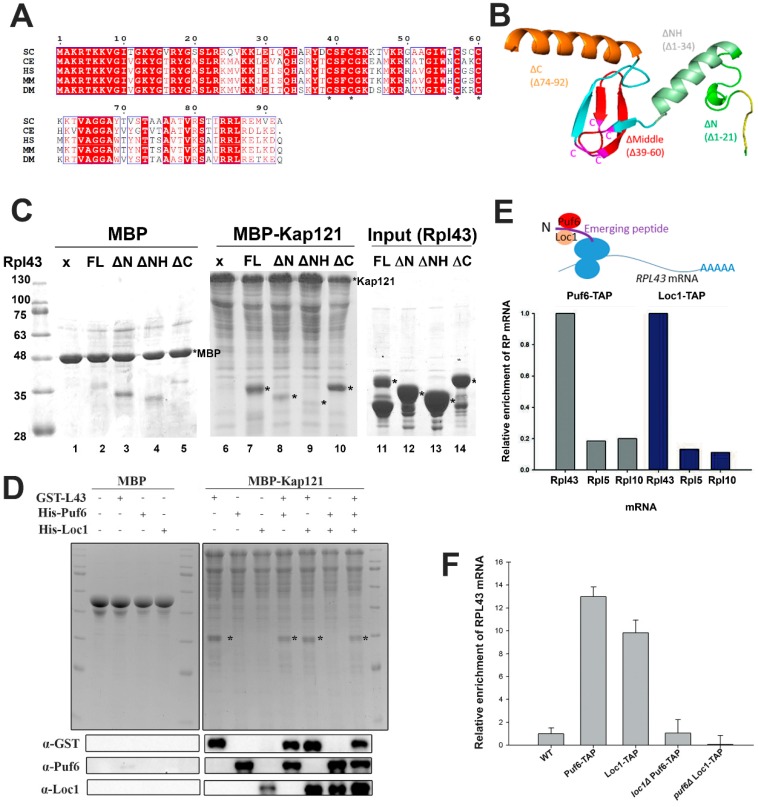
Puf6 and Loc1 interact with nascent Rpl43. (**A**) The Rpl43 sequences obtained from different species were aligned using ClustalW. SC: *Saccharomyces cerevisiae* (NP_015368.1); CE: *Caenorhabditis elegans* (NP_496957.1); HS: *Homo sapiens* (NP_000989.1); MM: *Mus musculus* (NP_033110.1); DM: *Drosophila melanogaster* (NP_723060.1). The conserved four cysteines are labelled with asterisks (*). (**B**) Rpl43 structure was adapted from PDB: 4V7F Chain k [46]. Each region generated for *rpl43* mutants is shown. (**C**) Recombinant maltose-binding protein (MBP) and MBP-Kap121 (PKL661) proteins were immobilized on amylose resins and tested for interactions with GST-Rpl43 (PKL474), GST-Rpl43ΔN (PKL584), GST-Rpl43ΔC (PKL585), and GST-Rpl43ΔNH (PKL652). (**D**) The *in vitro* interactions were examined in MBP and MBP-Kap121 (PKL661) with Puf6-His_6_ (PKL56), Loc1-His_6_ (PKL586), and GST-Rpl43 (PKL474). (**E**,**F**) *PUF6-TAP* (KLY134), *LOC1-TAP* (KLY475), *loc1*Δ*PUF6-TAP* (KLY415), and *puf6*Δ*LOC1-TAP* (KLY842) strains were treated with 0.2 mg/mL cycloheximide for 30 min and applied in the immunoprecipitation assay. The associated RNAs were extracted and quantitated by qPCR.

Rpl43 is a highly conserved protein. Several Rpl43 sequences were chosen from several model organisms for comparison, including yeast (*Saccharomyces cerevisiae*), nematode (*Caenorhabditis elegans*), human (*Homo sapiens*), mouse (*Mus musculus*), and fly (*Drosophila melanogaster*). The yeast Rpl43 sequence shares about 70% identity with other sequences. For instance, yeast Rpl43 shares 66.3% identity and 79.3% similarity with human Rpl43 (Figure 3A).

The N-terminus of Rpl43 is a random coil followed by an alpha helix, a conserved C4-type Zn finger domain composed of four beta-sheets, and then another alpha helix (Figure 3B adapted from PDB: 4V7F [46]). Zn finger domains are relatively small protein motifs important for interactions with DNA, RNA, proteins, or even lipids. Four conserved cysteines can chelate metals, such as Zn or Fe, or not bind ions. This motif is very important for the structure and functions of proteins. Four cysteines at amino acids 9, 42, 57, and 60 in Rpl43 form a C4-type Zn finger domain and are conserved across different species (Figure 3A,B).

Ribosomal proteins are translated in the cytoplasm and are required to be imported into the nucleus for assembly with rRNAs. Karyopherins are a group of proteins which play a central role in nucleocytoplasmic transport [47]. Some dedicated chaperones have been shown to be important for the ribosomal proteins to associate or disassociate with the karyopherins [14]. Kap121 is one of the major karyopherins responsible for the transport of ribosomal proteins [48]. Kap121 was fused with maltose-binding protein (MBP) and tested for its interaction with Rpl43 (Figure 3C). Kap121 can interact with Rpl43 directly. Deletion of the N-terminal coil (Δaa1-21) decreased the interaction with Kap121 (Figure 3C, lane 8). Further deletion of the N-terminal helix (Δaa1-34) ceased the interaction with Kap121 (Figure 3C, lane 9). However, deletion of the C-terminal helix (Δaa74-92) did not change the interaction with Kap (Figure 3C, lane 10). Therefore, the N-terminus is important for Rpl43 to interact with Kaps. Puf6, Loc1, and Rpl43 were added separately or in combination for binding assays with Kap121 to test whether Puf6 or Loc1 was required for the interaction or dissociation of Rpl43 with Kap. Puf6, Loc1, and Rpl43 can interact with Kap individually, and the addition of Puf6 or Loc1 did not change the interaction of Rpl43 with Kap (Figure 3D).

Dedicated chaperones may interact with nascent ribosomal proteins when they have just been translated. In this situation, the dedicated chaperones recognize the N-terminus and capture nascent ribosomal proteins co-translationally [32]. The dedicated chaperones can then pull down the mRNA of its associated ribosomal protein (Figure 3E, upper panel). To test this idea, cells were treated with cycloheximide to halt translation, and Puf6 and Loc1 were applied to test whether they can interact with translating *RPL43* mRNA. Compared with the controls, *RPL5* and *RPL10* mRNAs, Puf6 and Loc1 showed specificity toward *RPL43* mRNA (Figure 3E). Since Puf6, Loc1, and Rpl43 have been shown to form a trimer [38], we further tested this association in *puf6*Δ or *loc1*Δ mutants and found that mutation of either one significantly dropped the interaction with *RPL43* mRNA (Figure 3F). The results thus far imply that Puf6 and Loc1 can associate with nascent Rpl43 in a cooperative manner and are not required for the Kap interaction or release.

### 3.4. Determination of the Potential Interaction Sites among the Three Proteins

**Figure 4:**
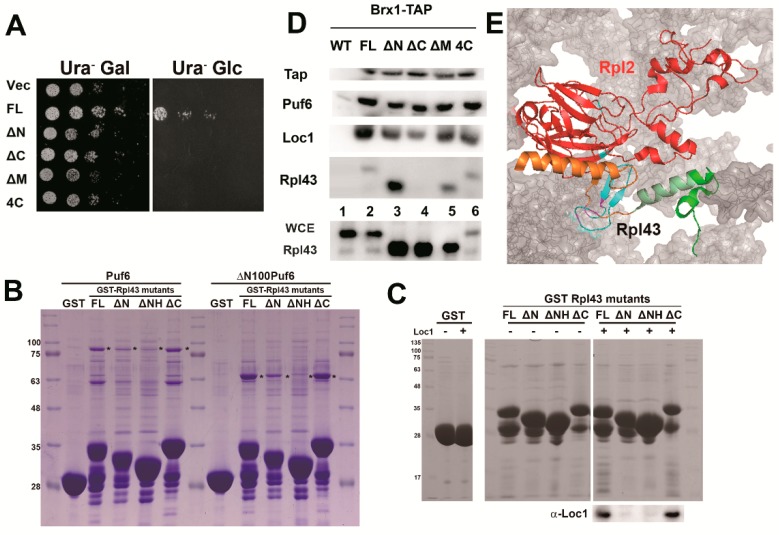
Examinations of Rpl43 mutants. (**A**) The growth assays of *RPL43-HA* (PKL350) and Rpl43 mutants, *rpl43*Δ*N-HA* (PKL657), *rpl43*Δ*C-HA* (PKL658), *rpl43*Δ*M-HA* (PKL659), and *rpl43-4C-HA* (PKL660), in the *GAL::RPL43* (KLY628) strain. Cells were normalized and spotted on Ura^-^Gal and Ura^-^Glc plates with serial dilution. (**B**,**C**) GST, GST-Rpl43 (PKL474), GST-Rpl43ΔN (PKL584), GST-Rpl43ΔC (PKL585), and GST-Rpl43ΔNH (PKL652) were used to interact with Puf6-His_6_ (PKL56), ΔN100Puf6-His_6_ (PKL512), and Loc1-His_6_ (PKL586). (**D**) Immunoprecipitation of Brx1-TAP with the Rpl43 mutants. (**E**) The Rpl43 (color depicted as in Figure 3B) and Rpl2 (orange) are shown on the pre-60S (Gray). PDB: 4V7F.

Several mutants were constructed to further dissect the interaction domains among the three proteins. The Rpl43 mutants described above (Figure 3B), deletion of the N-terminal coil (∆N: ∆1-21), deletion of the N-terminal helix (ΔNH: Δ1-34), deletion of the middle (∆M: ∆39-60), deletion of the C-terminal helix (∆C: ∆74-92), and mutation of the four conserved cysteines (4C) were used to determine the potential interaction site of Rpl43 with Pu6 and Loc1. All of these mutants could not support the growth of yeast (Figure 4A). To carry out the in vitro interaction assay, Rpl43 proteins with GST (glutathione S-transferase) fusion were used to examine the interaction with His_6_-tagged Puf6, ΔN100Puf6 (PUF domain only), and Loc1 (Figure 4B,C). Because the molecular weight of recombinant Loc1 is a similar size to that of GST-Rpl43, the signals could only be detected by Western blotting. The data revealed that FL and rpl43ΔC can interact with Puf6 (Figure 4B) and Loc1 (Figure 4C), but deletion of the N-terminus (rpl43ΔN and rpl43ΔNH) destroyed this interaction (Figure 4B,C). This is consistent with the observation that Puf6 and Loc1 can capture nascent Rpl43 co-translationally (Figure 3E): once the N-terminus emerges from the polypeptide exit tunnel (PET) of the ribosome, the dedicated chaperone loads and protects its accompanying ribosomal protein immediately [14]. Next, we tested whether these mutants could be incorporated into 60S subunits by using Brx1 to immunoprecipitate pre-60S. All of the mutants, except rpl43ΔC, could be found in 60S (Figure 4D). The C-terminal helix is buried in 60S and interacts with Rpl2 (Figure 4E). Therefore, the N-terminus is important for the associations of Rpl43 with Puf6 and Loc1, and the C-terminal helix is important for its interaction with 60S.

**Figure 5:**
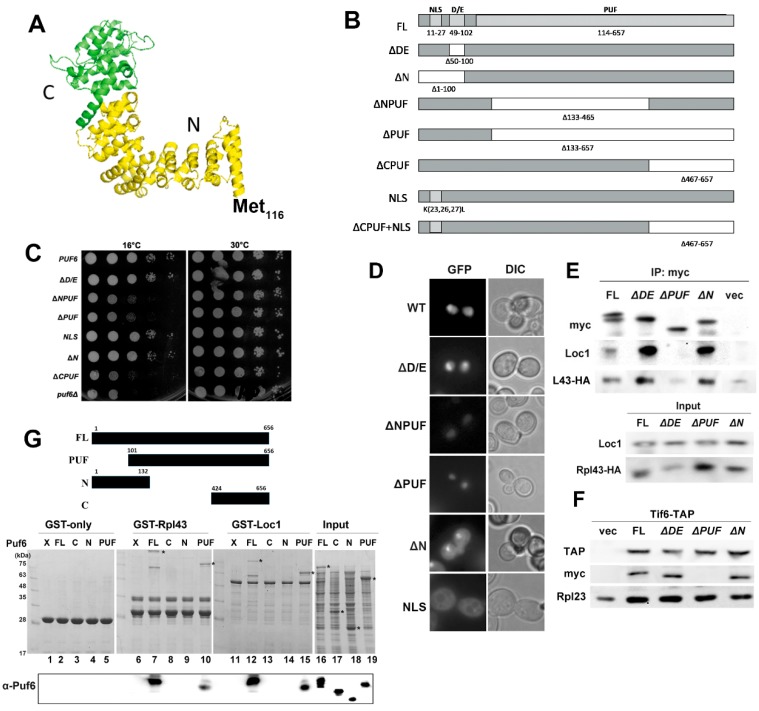
Examinations of Puf6 mutants. (**A**) The PUF domain of Puf6. PDB (4WZR) was used as a template in protein modeling. (**B**) A diagram of Puf6 truncation mutants. (**C**) *PUF6* (PKL52), *ΔD/E* (PKL53), *∆NPUF* (PKL55)*, ∆PUF* (PKL54)*, NLS* (PKL199), *∆N* (PKL188), Δ*CPUF* (PKL304), and vector were transformed to *puf6*Δ cells. Cells were normalized and spotted on a Leu^-^Glc plate with serial dilution. Plates were incubated at 16 and 30 °C. (**D**) The GFP localizations of various Puf6 mutants were monitored under fluorescence microscopy. (**E**) *PUF6-myc* (PKL85), *puf6*Δ*DE-myc* (PKL86), *puf6*Δ*PUF-myc* (PKL87), and *puf6ΔN-myc* (PKL189) were transformed to BY4741 containing *RPL43-HA* (PKL350). Puf6 was immunoprecipitated, and we examined the interaction with Loc1 and Rpl43. (**F**) Tif6-TAP was immunoprecipitated, and the interactions with Puf6 mutants were examined. (**G**) GST, GST-Rpl43 (PKL474), and GST-Loc1 (PKL400) interacted with Puf6 (PKL56), Puf6N (PKL662), Puf6C (PKL663), and the PUF domain (PKL512). The positions of Puf6 mutants were indicated with *.

Puf6 belongs to the PUF family of RNA-binding proteins, which are post-transcriptional regulators. Nearly all PUF homology domains contain eight Pumilio (PUM) repeats (~36 amino acids) that form an α-helical repeat rainbow-like structure [49,50,51]. Uniquely different from these proteins, Puf6 and its human homolog, Puf-A, contain 11 PUM repeats and assemble in an L shape. (Figure 5A—protein modeling with PDB (4WZR) as a template; the PUF domain of Puf6 spans from Met116 to the end of the sequence) [52]. The N-terminal 115 amino acids of Puf6 are not included in the PUF domain. It is a region rich with glutamic acids and aspartic acids (D/E) with an unidentified functional role and nuclear localization signal (NLS) to drive Puf6 into the nucleus (Figure 5B). Several Puf6 mutants, including ∆DE region, ∆N, ∆NPUF, ∆PUF, ∆CPUF, and NLS mutants, were constructed. The mutants and wild-type Puf6 were transformed into the *puf6*∆ strain for growth tests (Figure 5C). While the wild-type, ∆D/E, NLS, and ∆N mutants grew well, ∆NPUF, ∆PUF, and ∆CPUF did not complement the growth of *puf6*∆ cells (Figure 5C).

We monitored the distribution of different variations of Puf6 mutants in the cells (Figure 5D). Wild-type and ∆D/E mutants were mainly localized in the nucleus and nucleolus. ∆N and NLS were mostly distributed in the cytoplasm, implying that this is genuine NLS signal. However, while they still can complement the growth of *puf6*∆ cells (Figure 5C), this suggests the redundancy of NLS. ∆NPUF and ∆PUF lost their nucleolar enhancement and localized only in the nucleus, suggesting that the PUF domain is important in ribosome synthesis (Figure 5D). ∆D/E, ∆PUF, and ∆N were used to demonstrate interactions in vivo, and only ∆PUF lost its interaction with Loc1, Rpl43, and 60S (Figure 5E,F). An intact PUF domain is required to interact with Rpl43 and Loc1 in vitro (Figure 5G, lanes 10 and 15). The N-terminus (Figure 5G, lanes 9 and 14) or partial PUF domain (Figure 5G, lanes 8 and 13) did not support these interactions. These findings support that the PUF domain is required for Puf6 to form a ternary complex with Rpl43 and Loc1 and to interact with pre-60S.

**Figure 6:**
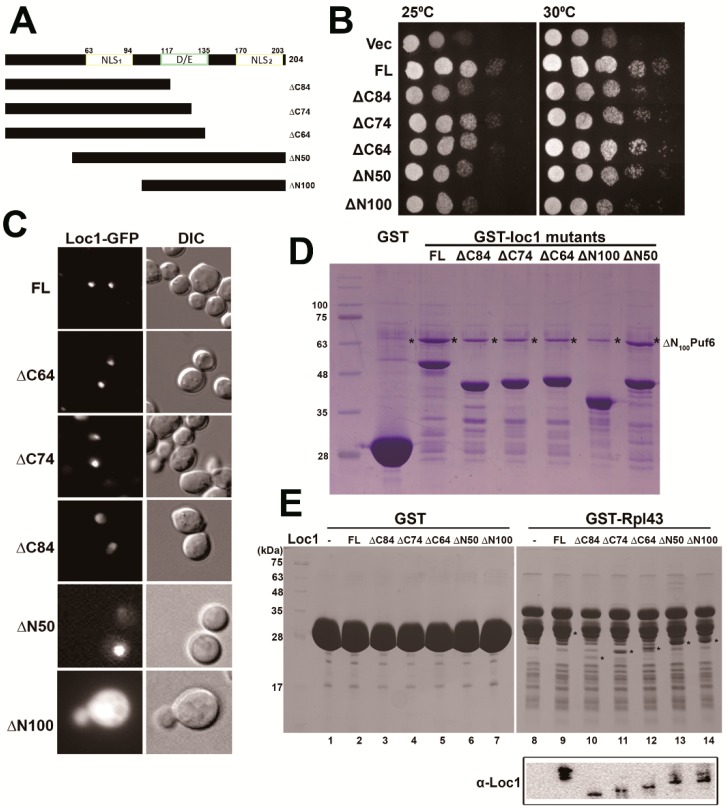
Examinations of Loc1 mutants. (**A**) A diagram of the Loc1 truncation mutants. (**B**) *LOC1-GFP* (PKL337), *loc1*Δ*C84-GFP* (PKL449), *loc1*Δ*C74-GFP* (PKL450), *loc1*Δ*C64-GFP* (PKL451), *loc1*Δ*N50-GFP* (PKL655), and *loc1*Δ*N100-GFP* (PKL518) were transformed to *loc1*Δ (KLY218) and assayed via a growth test. (**C**) The localizations of Loc1 mutants were monitored using fluorescence microscopy. (**D**) GST, GST-Loc1 (PKL400), GST-Loc1ΔC84 (PKL580), GST-Loc1ΔC74 (PKL581), GST-Loc1ΔC64 (PKL653), GST-Loc1ΔN50 (PKL583), and GST-Loc1ΔN100 (PKL582) interacted with Puf6. (**E**) GST and GST-Rpl43 (PKL474) interacted with Loc1 (PKL586), Loc1ΔC84 (PKL588), Loc1ΔC74 (PKL589), Loc1ΔC64 (PKL592), Loc1ΔN50 (PKL650), and Loc1ΔN100 (PKL592). The positions of Loc1 mutants were indicated with *.

Preliminary sequence analysis revealed that Loc1 does not contain an obvious functional domain, and its structure is not yet solved. Sequence analysis also indicated that two potential NLSs could be found at the N- and C-termini and one short stretch of the DE-rich region of the protein (Figure 6A). The NLS1, ranging over amino acids 63–94, is similar to a classical bipartite NLS sequence, i.e., KKDKKGKYSEKDLNIPTLNRAIVP GVKIRRGK, revealing that two clusters of basic amino acids are separated by a 10–12-amino-acid linker [53]. We constructed N- and C-terminal truncation variants of Loc1 proteins for functional studies, i.e., ∆N50 deleted the first 50 amino acids, ∆N100 further deleted to eliminate NLS1, ∆C64 deleted the NLS2, ∆C74 deleted the NLS2 and partial D/E region, and ∆C84 deleted the NLS2 and the whole D/E region (Figure 6A). Loc1 mutant plasmids were first transformed into the *loc1*∆ strain for complementation assays (Figure 6B). Deletion of 64 amino acids from the C-terminus (loc1∆C64) did not impair the growth of Loc1, but further deletion of 10 or 20 more amino acids (loc1∆C74 and loc1∆C84) decreased cell growth (Figure 6B). Deletion of 50 (loc1∆N50) and 100 (loc1∆N100) amino acids from the N-terminus impaired growth slightly and significantly, respectively (Figure 6B). Next, we observed the localization of these loc1 mutants (Figure 6C). Loc1 was predominantly localized in the nucleolus. Interestingly, Loc1 mutants with longer C-terminal truncations showed stronger signals in the nucleoplasm: Loc1∆C64 was present only in the nucleolus, loc1∆C74 was localized predominantly in the nucleolus and slightly in the nucleoplasm, and loc1∆C84 was distributed only in the nucleus. Loc1∆N50 was localized in the nucleus, but loc1∆N100 was localized in the cytoplasm. Therefore, NLS1 seems to be the major sequence driving Loc1 into the nucleus. 

We tested the interactions between the Loc1 mutants and Puf6 and Rpl43 (Figure 6D,E). While loc1∆N50 maintained the interaction with ΔN100Puf6, mutants loc1∆N100, loc1∆C64, loc1∆C74, and loc1∆C84 decreased the interaction with Puf6 (Figure 6D). All the Loc1 mutants dropped the interaction with Rpl43; however, the C-terminal truncation gave a more significant decrease (Figure 6E). Therefore, Loc1 may use its C-terminus to interact with Puf6 and Rpl43.

## 4. Discussion

Rpl43 is a small ribosomal protein (about 10 KDa) with only 92 amino acids; it contains a conserved C4-type Zn finger domain made up of four beta-sheets at the center and two helices connected at the N- and C-termini. We found that Rpl43 contains Zn at a 1:1 ratio. Mutations of four conserved cysteines to serine can abolish the chelation of Zn (unpublished data). Although the rpl43(4C) mutant could still be incorporated into pre-60S, it did not complement growth. The half-life of Rpl43 was very short and decreased by 50% within 10 min after translation arrest. This instability was even more severe in *puf6*Δ and *loc1*Δ mutants (Figure 2).

Ribosomal proteins are very unstable because of their unique structural characteristics, which include highly positive charges and unstructured extensions. Many dedicated chaperones have been identified to protect the stability of ribosomal proteins, such as Sqt1 with Rpl10 [16,17], Acl4 with Rpl4 [18,19], Yar1 with Rps3 [20,21], Rrb1 with Rpl3 [22,23], Tsr2 with Rps26 [24,25], Syo1 with Rpl5 and Rpl11 [26,27,28], Bcp1 with Rpl23 [29], Nap1 with Rps6 [30], and Tsr4 with Rps2 [30,31] These chaperones may associate with nascent ribosomal proteins co-translationally and accompany the transport process to the assembly point [32]. They may also have additional functions in regulation. For example, Syo1 can synchronize the transport of Rpl5 and Rpl11 and associate them with 5S rRNA to achieve the correct orientation [26,27,28]. Tsr2 and Bcp1 can dissociate ribosomal proteins from karyopherins in a RanGTP-independent manner [24,25,29] (see reviews in [14,33]).

RNA-binding proteins have been shown to be important in ribosome biogenesis and development [54,55,56,57]. Puf6 and Loc1 have been shown to regulate the cellular distribution of *ASH1* mRNA. The loss of these two proteins decreases the levels of 60S subunits. However, the molecular functions of Puf6 and Loc1 in 60S biogenesis remain unclear. We have shown that these proteins can form a hetero-trimer with Rpl43 and are critical to maintaining the protein level of Rpl43 and its assembly to pre-60S subunits [38]. Here we provide further evidence that Puf6 and Loc1 are important to maintaining the protein stability of Rpl43 (Figure 2). The N-terminal 21 amino acids of Rpl43 are important for Kap, Puf6, and Loc1 interactions (see the rpl43ΔN mutant in Figure 3 and Figure 4). This region is less structured (Figure 3B) and may be more easily targeted for degradation than other regions. We also demonstrated that Puf6 and Loc1 can bind nascent Rpl43 co-translationally in a cooperative manner (Figure 3). Therefore, once the N-terminus of Rpl43 emerges from the PET site, Puf6 and Loc1 may majorly associate with this region and protect its stability.

The present study supports the supposition that Puf6 and Loc1 are associated with nascent Rpl43 for transport but do not depend on Rpl43 for import [38]. While Puf6 lost its PUF domain to interact with Loc1, Rpl43, or 60S, the ΔPUF mutant could still be localized in the nucleus (Figure 5). A similar finding was observed in Loc1 mutants. Truncations of the N- or C-terminus of Loc1 decreased its interactions with Puf6 or Rpl43 but were still retained in the nucleus (Figure 6). This situation is likely because Puf6 and Loc1 have additional functions with *ASH1* mRNA in the nucleus and could therefore be transported with Rpl43 or as individual proteins.

The loss of Rpl43 did not influence the loading of Puf6 or Loc1 but affected their release [38]. Similarly, although deletion of *puf6* affected the interaction of Loc1 with nascent Rpl43 (Figure 3), Loc1 could still be loaded onto 60S [38]. However, the protein could not be released properly [38]. The positions of Puf6 and Loc1 on pre-60S have not been solved, but our data suggest that they are bound in the vicinity of Rpl43. This supposition supports the hypothesis that trimer formation is important for the accuracy of this local domain. Only when Rpl43, Puf6, and Loc1 are loaded as a ternary complex onto pre-60S can the process to the next step begin properly.

## Figures and Tables

**Table 1:** Strains used in this study.

Strain	Genotype	Source
BY4741	*MATa his3Δ1 leu2Δ0 met15Δ0 ura3Δ0*	
KLY67	*MATa his3Δ1 leu2Δ0 met15Δ0 ura3Δ0 puf6*Δ*::KanMX*	[38]
KLY218	*MATa his3Δ1 leu2Δ0 met15Δ0 ura3Δ0 loc1*Δ*::KanMX*	[38]
KLY312	*his3Δ1 leu2Δ0 met15Δ0 ura3Δ0 puf6∆::KanMX loc1∆::KanMX*	[38]
KLY134	*MATa PUF6-TAP::HIS3MX his3Δ1 leu2Δ0 met15Δ0 ura3Δ0*	This study
KLY317	*MATa ARX1-TAP::HIS3MX his3Δ1 leu2Δ0 met15Δ0 ura3Δ0*	[38]
KLY415	*MATa loc1*Δ*::KanMX PUF6-TAP::HIS3MX his3Δ1 leu2Δ0 met15Δ0 ura3Δ0*	This study
KLY471	*MATa BRX1-TAP::HIS3MX his3Δ1 leu2Δ0 met15Δ0 ura3Δ0*	[38]
KLY475	*MATa LOC1-TAP::HIS3MX his3Δ1 leu2Δ0 met15Δ0 ura3Δ0*	[38]
KLY583	*MATa TIF6-TAP::HIS3MX his3Δ1 leu2Δ0 met15Δ0 ura3Δ0*	[38]
KLY596	*MATa RIX1-TAP::HIS3MX his3Δ1 leu2Δ0 met15Δ0 ura3Δ0*	[38]
KLY598	*MATa SSF1-TAP::HIS3MX his3Δ1 leu2Δ0 met15Δ0 ura3Δ0*	[38]
KLY628	*MATa his3Δ1 leu2Δ0 met15Δ0 ura3Δ0 rpl43a∆::KanMX rpl43b∆::CloNAT GAL::RPL43B-HA (PKL381 CEN HIS3)*	[38]
KLY672	*MATa REI1-TAP::HIS3MX his3Δ1 leu2Δ0 met15Δ0 ura3Δ0*	This study
KLY816	*MATa NOC2-TAP::HIS3MX his3Δ1 leu2Δ0 met15Δ0 ura3Δ0*	This study
KLY822	*MATa CIC1-TAP::HIS3MX his3Δ1 leu2Δ0 met15Δ0 ura3Δ0*	This study
KLY842	*MATa puf6Δ::KanMX LOC-TAP::HIS3MX his3Δ1 leu2Δ0 met15Δ0 ura3Δ0*	This study
KLY875	*MATa NOP15-TAP::HIS3MX his3Δ1 leu2Δ0 met15Δ0 ura3Δ0*	This study
KLY876	*MATa NOP56-TAP::HIS3MX his3Δ1 leu2Δ0 met15Δ0 ura3Δ0*	This study
KLY877	*MATa NOC4-TAP::HIS3MX his3Δ1 leu2Δ0 met15Δ0 ura3Δ0*	This study

**Table 2:** Plasmids used in this study.

Plasmid	Gene	Relevant Marker	Source
PKL52	*PUF6-GFP*	*CEN LEU2*	This study
PKL53	*puf6*Δ*DE-GFP*	*CEN LEU2*	This study
PKL54	*puf6*Δ*PUF-GFP*	*CEN LEU2*	This study
PKL55	*puf6*Δ*NPUF-GFP*	*CEN LEU2*	[38]
PKL56	*pET28a-PUF6*	Kan	[38]
PKL85	*PUF6-myc*	*CEN LEU2*	[38]
PKL86	*puf6ΔD/E-myc*	*CEN LEU2*	This study
PKL87	*puf6ΔPUF-myc*	*CEN LEU2*	This study
PKL88	*puf6ΔNPUF -myc*	*CEN LEU2*	This study
PKL188	*puf6*Δ*N-GFP*	*CEN LEU2*	This study
PKL189	*puf6ΔN-myc*	*CEN LEU2*	This study
PKL198	*puf6(NLS)-myc*	*CEN LEU2*	This study
PKL199	*puf6(NLS)-GFP*	*CEN LEU2*	This study
PKL288	*pET28a-LOC1*	Kan	[38]
PKL306	*puf6ΔCPUF-myc*	*CEN LEU2*	This study
PKL333	*PUF6*	*2*μ *URA3*	This study
PKL337	*LOC1-GFP*	*CEN LEU2*	[38]
PKL349	*RPL43A-HA*	*CEN URA3*	This study
PKL350	*RPL43B-HA*	*CEN URA3*	[38]
PKL400	*GST-LOC1*	*CEN LEU2*	This study
PKL449	*loc1*Δ*C84-GFP*	*CEN LEU2*	This study
PKL450	*loc1*Δ*C74-GFP*	*CEN LEU2*	This study
PKL451	*loc1*Δ*C64-GFP*	*CEN LEU2*	This study
PKL474	*GST-RPL43*	Amp	[38]
PKL512	*pET28a-puf6(PUF)*	Kan	This study
PKL518	*loc1*Δ*N100-GFP*	*CEN LEU2*	This study
PKL563	*RPL43B-HA*	*CEN HIS3*	This study
PKL580	*GST-loc1*Δ*C84*	Amp	This study
PKL581	*GST-loc1*Δ*C74*	Amp	This study
PKL582	*GST-loc1ΔN100*	Amp	This study
PKL583	*GST-loc1ΔN50*	Amp	This study
PKL584	*GST-L43ΔC helix*	Amp	This study
PKL585	*GST-L43ΔN coil*	Amp	This study
PKL586	*pET28a-loc1*Δ*C84*	Kan	This study
PKL588	*pET28a-loc1*Δ*C74*	Kan	This study
PKL590	*pET28a-loc1*Δ*C64*	Kan	This study
PKL592	*pET28a-loc1*Δ*N100*	Kan	This study
PKL650	*pET28a-loc1*Δ*N50*	Kan	This study
PKL652	*GST-L43*Δ*N helix*	Amp	This study
PKL653	*GST-loc1ΔC64*	Amp	This study
PKL655	*loc1ΔN50-GFP*	*CEN LEU2*	This study
PKL657	*rpl43BΔN-HA*	*CEN URA3*	This study
PKL658	*rpl43BΔC-HA*	*CEN URA3*	This study
PKL659	*rpl43BΔM-HA*	*CEN URA3*	This study
PKL660	*rpl43B-4C-HA*	*CEN URA3*	This study
PKL661	*MBP-KAP121*	Amp	This study
PKL662	*pET28a-puf6-Nter*	Kan	This study
PKL663	*pET28a-puf6-Cter*	Kan	This study
PKL746	*LOC1*	*2*μ *LEU2*	This study

**Table 3:** Oligos.

Oligo#		Sequence	Source
probes			
#1 (KLO48)	5S	TCTGGTAGATATGGCCGCAACC	[39]
#2 (KLO49)	18S	GTCTGGACCTGGTGAGTTTCCC	[39]
#3 (KLO50)	20S	TCTTGCCCAGTAAAAGCTCTCATGC	[39]
#4 (KLO51)	23S	TGTTACCTCTGGGCCCCGATTG	[39]
#5 (KLO52)	35S, 27SA	TCCAGTTACGAAAATTCTTGTTTTTGACAA	[39]
#6 (KLO53)	5.8S	CGCTGCGTTCTTCATCGATGCG	[39]
#7 (KLO54_	7S	GGCCAGCAATTTCAAGTTA	[39]
#9 (KLO56)	25S	CCCGCCGTTTACCCGCGCTTGG	[39]
**qPCR Primers**			
KLO323	*RPL5*	TAGCTGCTGCCTACTCCCACGA	[32]
KLO324	*RPL5*	GCAGCAGCCCAGTTGGTCAAA	[32]
KLO325	*RPL10*	TGTCTTGTGCCGGTGCGGAT	[32]
KLO326	*RPL10*	TGTCGACACGAGCGGCCAAA	[32]
KLO327	*RPL43*	CAAGAGAGGTGCTGCTGGTA	This study
KLO328	*RPL43*	GCAGTGGAGACAGTGTAAGCA	This study
